# Piperine as Therapeutic Agent in Paracetamol-Induced Hepatotoxicity in Mice

**DOI:** 10.3390/pharmaceutics14091800

**Published:** 2022-08-26

**Authors:** Aline Meireles Coelho, Isabela Ferreira Queiroz, Luiza Oliveira Perucci, Melina Oliveira de Souza, Wanderson Geraldo Lima, André Talvani, Daniela Caldeira Costa

**Affiliations:** 1 Department of Biological Sciences (DECBI), Institute of Exact and Biological Sciences, Federal University of Ouro Preto (UFOP), Ouro Preto 35400-000, Brazil; 2 Center for Research in Biological Sciences (NUPEB), Federal University of Ouro Preto (UFOP), Ouro Preto 35400-000, Brazil; 3 Department of Food (DEALI), School of Nutrition, Federal University of Ouro Preto (UFOP), Ouro Preto 35400-000, Brazil

**Keywords:** paracetamol, acetaminophen, piperine, redox status, inflamassome

## Abstract

High doses of paracetamol (APAP) can cause irreversible liver damage. Piperine (P) inhibits cytochrome P450, which is involved in the metabolism of various xenobiotics, including paracetamol. We evaluated the hepatoprotective effects of piperine with or without N-acetylcysteine (NAC) in APAP-induced hepatotoxicity. The mice were treated with two doses of piperine (P20 or P40) and/or NAC at 2 h after administration of APAP. The NAC+P20 and NAC+P40 groups showed a reduced area of necrosis, MMP-9 activity, and *Casp-1* expression. Furthermore, the NAC+P20 group was the only treatment that reduced alanine aminotransferase (ALT) and increased the levels of sulfhydryl groups (-SH). In the NAC+P40 group, *NLRP-3* expression was reduced. Aspartate aminotransferase (AST), thiobarbituric acid-reactive substances (TBARS), and IL-1β expression decreased in the NAC, NAC+P20, and NAC+P40 groups compared to the APAP group. The liver necrosis area, TNF levels, carbonylated protein, and IL-18 expression decreased in the P40, NAC, NAC+P20, and NAC+P40 groups compared to the APAP group. The cytokine IL-6 was reduced in all treatments. Piperine can be used in combination with NAC to treat APAP-induced hepatotoxicity.

## 1. Introduction

The drug acetaminophen (N-acetyl-ρ-aminophen, APAP), also known as paracetamol, is widely used worldwide for its analgesic and antipyretic properties. However, APAP intoxication induces necrosis in the centrilobular region of the liver, causing acute liver failure (ALI) and possibly death [1].

When administered, paracetamol is metabolized by about 10% to 20% by the cytochrome P450 (CYP) pathway where the CYP2E1 isoenzyme contributes the most and, to a lesser extent, the CYP1A2 and CYP3A4 isoenzymes also contribute. In this oxidation step, the toxic adduct N-acetyl-p-benzo-quinone imine (NAPQI) is formed [2,3,4,5].

In therapeutic doses, the metabolite NAPQI can be easily detoxified by glutathione (GSH). However, excessive doses of APAP cause glutathione depletion, and the toxic metabolite binds to cellular proteins, especially in the mitochondria, inducing the formation of reactive species [3,6].

N-acetylcysteine (NAC) has been used for the treatment of paracetamol intoxication since the 1970s. The therapeutic effect of NAC is due to its capacity to stimulate the synthesis of reduced glutathione, the concentration of which in cells decreases in the presence of APAP. However, NAC has significant side effects such as nausea, vomiting, bronchospasm, and anaphylaxis, and there is still some uncertainty regarding the dose to be administered and the duration of treatment [4,7,8,9].

Black pepper (*Piper nigrum*) belongs to the Piperaceae family and is widely used in cooking because of its characteristic flavor. It has also been used as a preservative, perfume, food additive, and traditional medicine, mainly to treat epilepsy and headaches, among other conditions [10,11]. Its therapeutic effects are attributed to piperine (1-(5-[1,3-benzodioxol-5-yl]-1-oxo-2,4-pentadienyl) piperidine), an organic alkaloid with a molecular weight of 285 g/mol [12].

Some therapeutic compounds reported in the literature for their low bioavailability were associated with piperine and demonstrated an increase in bioavailability and the ability to potentiate protective effects [13]. This characteristic of piperine enhanced the properties of curcumin [14], phyllanthin [15], quercertin [16], resveratrol [17], and other compounds.

Previous studies show that piperine is a selective inhibitor of the cytochrome P450 2E1 complex, which is involved in the metabolism of different xenobiotics, including paracetamol, and has hepatoprotective effects [18,19].

Piperine has several biological functions, including anti-inflammatory properties, and plays an important role in the inhibition of TNF and IL-6, which are pro-inflammatory cytokines responsible for the initiation of the inflammatory response [20,21]. Furthermore, it has an antioxidant activity, which results in reduced levels of malondialdehyde (MDA), an aldehyde resulting from lipid peroxidation [22].

Thus, based on the actions of piperine (i.e., as a cytochrome P450 inhibitor, antioxidant, and anti-inflammatory agent) and the fact that APAP at toxic doses is metabolized by cytochrome P450, generating oxidative and inflammatory stress, we hypothesize that piperine may reduce redox and inflammatory imbalance and, consequently, inhibit the mechanisms responsible for hepatocyte necrosis. Thus, the aim of this study was to evaluate the hepatoprotective effects of piperine in combination with NAC in the treatment of liver injury induced by APAP.

## 2. Materials and Methods

### 2.1. Animals

C57BL/6 male mice with an average weight of 23 to 25 g were used. The animals were purchased from the Animal Science Center of the Federal University of Ouro Preto (UFOP), Brazil, and housed at a controlled temperature (24 ± 1 °C), with 12-h light/dark cycles and ad libitum feed. The study was approved by the Ethics Committee on Animal Use of UFOP (protocol number 5402121118).

### 2.2. Experimental Design

The animals were fasted for 6 h before receiving paracetamol (500 mg/kg) or vehicle (distilled water) by orogastric gavage. The dose used to develop the APAP-induced liver injury model has been described in the literature [23,24] and standardized by the laboratory team [25,26,27]. Two hours after APAP administration, we performed the treatments with 300 mg/kg of N-acetylcysteine (NAC) and/or piperine dissolved in 0.5% carboxymethylcellulose at doses of 20 mg/kg or 40 mg/kg. The control and APAP groups received the vehicle carboxymethylcellulose 0.5%. Euthanasia occurred 12 h after APAP intoxication. There was no weight loss or death in the animals at 12 h after overdose. Blood was collected for serum, and the liver was fractionated for histology and other analyses. The experimental groups were as follows: the control group (C; N = 7), paracetamol (APAP; N = 7), N-acetylcysteine (NAC; N = 7), piperine 20 mg/kg (P20; N = 7), piperine 40 mg/kg (P40; N = 7), piperine 20 mg/kg in combination with N-acetylcysteine (NAC+P20; N = 7), and piperine 40 mg/kg in combination with N-acetylcysteine (NAC+P40; N = 7). The number of animals in each group (N = 7) was selected using the BioEstat 5.3 program.

### 2.3. Liver Function

Serum alanine aminotransferase (ALT) and aspartate aminotransferase (AST) levels were measured using commercial kits from the LABTEST^®^ (Lagoa Santa, MG, Brazil) according to the manufacturer’s protocols.

### 2.4. Liver Histological Analysis

Liver fragments were preserved in buffered formalin (4%). Liver tissues were fixed by processing in an alcohol series (70–100%) and subsequently immersed in paraffin. Paraffin sections (4 μm) were obtained using a semi-automatic microtome and placed on histological slides. Sections were stained using the hematoxylin and eosin (H&E) technique. Photomicrographs of the liver were taken using a Leica optical microscope coupled to a DM5000 digital camera, at 40× magnification using the Leica Application Suite^®^ analysis software. Morphometric analysis was performed using ImageJ software (version 1.32j) to quantify the area of necrosis, hyperemia index, and binucleation of hepatocytes. Randomly obtained images were analyzed using ImageJ software to delimit and quantify the necrotic areas. Hepatocyte binucleation and hyperemia were evaluated using ImageJ software based on qualitative methods by summing the photomicrographs of the positive fields for the respective lesions.

### 2.5. Biomarkers of Damage Caused by Lipid and Protein Oxidation

To determine the dosages, liver homogenates (100 mg) were prepared in the presence of 1 mM protease inhibitor (PMSF). Oxidized lipids are biomarkers of lipid peroxidation and have affinity for thiobarbituric acid (TBA), allowing the detection of reactive species and performing thiobarbituric acid-reactive substances (TBARS) quantification at 532 nm absorbance as described by Draper et al. [28]. The oxidation of proteins by reactive oxygen species (ROS) induces the formation of carbonylated proteins, which are indicators of this oxidation. The levels of carbonylated proteins were determined using the method of Levine et al. [29]. The absorbance of the supernatant was read using a UV spectrophotometer at a wavelength of 370 nm. The data for both were expressed as nmol/mg protein.

### 2.6. Analysis of Antioxidant Defenses

Glutathione is an important molecule in the antioxidant defense of the liver upon APAP intoxication, which has a sulfhydryl (-SH) group in its structure. Sulfhydryl groups are known to decrease as oxidative stress increases [30].

The technique uses the thiol groups of the samples and reacts with 0.01 M DTNB (2-nitrobenzoic acid) in addition to methanol. For the standard curve, we performed serial dilutions of L-cysteine reagent dissolved in 0.02 M triethanolamine (TEA). The absorbance of the supernatant was read using a UV spectrophotometer (412 nm). The values were normalized to the total protein content of the samples.

### 2.7. Cytokine Analysis

The inflammatory cytokines TNF and IL-6 and the immunoregulatory cytokine IL-10 in serum were quantified by enzyme-linked immunosorbent assay (ELISA) using commercial kits from Peprotech ^®^ (Rocky Hill, NJ, USA and Biorbyte, Cambridge, UK).

### 2.8. Cytochrome P450 2E1 (CYP2E1) Assay

Cytochrome P450 2E1 (CYP2E1) was quantified in liver homogenates using an ELISA immunoassay kit for mouse samples. A CYP2E1-specific antibody was pre-coated onto a microplate. The samples and standard were pipetted into the wells and any CYP2E1 present was bound by the immobilized antibody. The unbound substances were washed away, and a biotin conjugate antibody specific for CYP2E1 was added to the wells. After washing, streptavidin A conjugated with horseradish peroxidase (HRP) was added to each well. Subsequently, the wells were washed to remove excess of streptavidin-enzyme conjugates. A substrate solution was added to the wells and color developed in proportion to the amount of CYP2E1 bound in the initial step. Color development was stopped, and the color intensity was measured by the colorimetric method (450 nm) (Abbkine, Wuhan, Hubei, China).

### 2.9. Real-Time Reverse Transcription PCR (qRT-PCR)

Total RNA was extracted from liver samples using Trizol^TM^ (Invitrogen, Carlbad, CA, USA) and chloroform reagents (Sigma-Aldrich, St. Louis, MO, USA). For RNA isolation, isopropanol was added to precipitate RNA and the samples were centrifuged at 12,000× *g* for 10 min at 4 °C. The RNA pellet was washed with ethanol (75%) and dissolved in milli Q water. The isolated RNA was quantified in a nanodrop (260 nm), and the purity indicator (260/280 nm) was evaluated. RNA integrity was analyzed by agarose gel electrophoresis. Synthesis of complementary deoxyribonucleic acid (cDNA) was performed using a quantity of the extracted total RNA (2 µg) and the Capacity cDNA reverse transcription kit (Thermo Fisher, Waltham, MA, USA). Diluted cDNA (1 μL), SYBR^®^ Green PCR Master Mix (5 μL) (Thermo Fisher, Waltham, MA, USA), and DNAse-free water (3 μL) were used for sample preparation. qRT-PCR amplification was performed using the Applied Biosystems ABI 7300 instrument. The expression of genes of interest was normalized to that of β-actin (Actb). The sequences of the primers used were as follows: CYP2E1 F′TTTCCCTAAGTATCCTCCGTGAC, R′TCGTAATCGAAGCGTTTGTTG; CYP1A2 F′ACAAGACCCAGAGCGAGAAG, R′GCAGCAGGATGGCTAAGAAG; NLRP3 F′GGCGAGACCTCTGGGAAAAA, R′CCAGCAAACCCATCCACTCT; Casp-1 F′CTGGGACCCTCAAGTTTTGCC, R′GGCAAGACGTGTACGAGTGGT; IL-1β F′AGAGCCCATCCTCTGTGACT, R′GGAGCCTGTAGGTGCAGTTGT; IL18 F′ATTTTACTATCCTTCACCGAGAGG, R′TGTTCGAGGATATGACTGATATTGA; β-actin F′CACTGTCGAGTCGCGTCCA, R′TCATCCATGGCGAACTGGTG. Expression level analyses were performed using the relative gene expression quantification method (comparative Cq or ΔCq). Upregulation and downregulation of genes of the inflammasome pathway and the isoenzymes (CYP2E1 and CYP1A2) of cytochrome P450 was determined using a heat map (http://www1.heatmapper.ca/expression/ accessed on 7 April 2022).

### 2.10. Zymography

To evaluate the activity of metalloproteinase 9 (MMP-9), we used the zymography technique [31,32]. The liver samples were prepared in the presence of a protease inhibitor. The samples were electrophoresed in polyacrylamide gels (8%) copolymerized with 2 mg/mL gelatin. Then, the gels were washed in Triton X-100 solution (2.5%) and incubated in an incubation buffer [50 mM Tris, 150 mM NaCl, 5 mM CaCl_2_ and 0.05% NaN_3_ (pH 7.5)] for 18 h at 37 °C. The gels were stained using Coomassie Brilliant Blue G-250 (0.05%) and subsequently destained in methanol (4%) in the presence of acetic acid solution (8%). Bands were detected using the optical density of each band, and densitometry was performed using the IMAGEJ version 1.32 software (National Institutes of Health, Bethesda, MD, USA).

### 2.11. Statistical Analyses

Data are represented as the mean and standard error of the mean. The results were analyzed using GraphPadPrism 8.0 (GraphPad Software Inc., San Diego, CA, USA) and the Kolmogorov–Smirnov test for normality. Statistical analysis was performed by one-way ANOVA with Tukey’s post-test. For the results that did not show a normal distribution, we used contingency analysis and Fisher’s exact test, and the data were expressed with a dot plot. Statistical significance was set at *p* < 0.05.

## 3. Results

**Figure 1:**
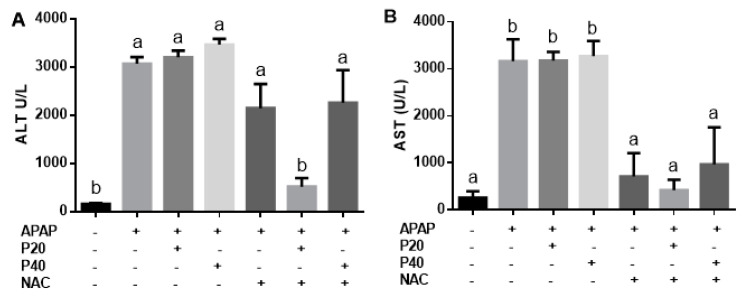
Evaluation of liver function by measuring ALT (**A**) and AST (**B**) concentration in C57BL/6 mice intoxicated with paracetamol (500 mg/kg). Statistical analysis was performed using one-way analysis of variance and Tukey’s post-test. Different letters (a, b) indicate significant difference between groups (*p* < 0.05), whereas the same letters indicate no significant difference between groups (*p* > 0.05). (+) Presence or (−) absence of paracetamol, piperine 20 mg/kg, piperine 40 mg/kg, N-acetylcysteine. ALT: alanine aminotransferase; AST: aspartate aminotransferase.

To evaluate liver function after intoxication, serum levels of ALT and AST were measured. A significant increase in ALT activity was observed 12 h after APAP intoxication (Figure 1A), and only the group treated with the combination of NAC and piperine 20 mg/kg showed a significant reduction in serum ALT. In the other experimental groups, no significant differences were observed compared to the APAP group. A reduction in AST was observed in the NAC, NAC plus piperine 20 mg/kg, and piperine 40 mg/kg groups compared to the APAP group (Figure 1B).

**Figure 2:**
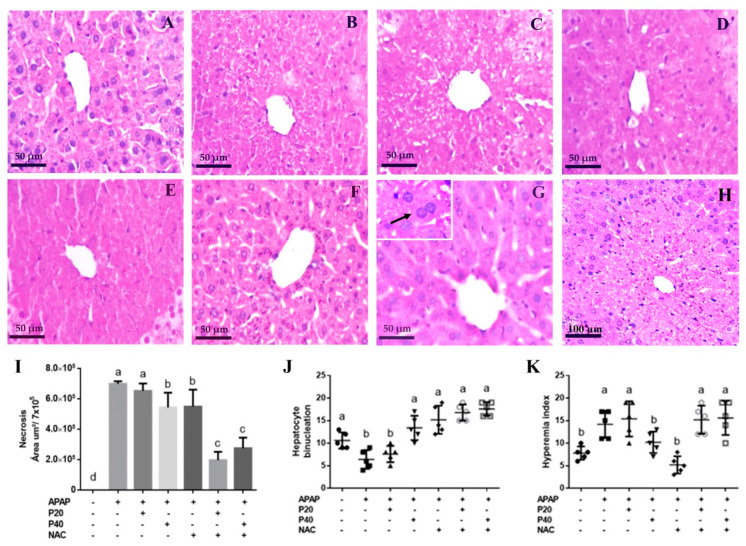
Representative photomicrographs, percentage of hepatic necrosis, and histopathological evaluation of the effects of paracetamol on liver function. Histological sections of the liver of C57BL/6 mice intoxicated with paracetamol (500 mg/kg) and treated after 2 h of intoxication with piperine and/or NAC. (**A**) The control group (40×), histological section showing normal architecture. (**B**) APAP group. (**C**,**D**) Groups treated with piperine 20 mg/kg and 40 mg/kg, respectively. (**E**–**G**) Groups treated with NAC and the combination of NAC with piperine 20 mg/kg or 40 mg/kg, respectively. (**G**) Representative insert with binucleated hepatocyte (indicated by black arrow). (**H**) Image of the APAP group at lowest magnification (20×). Staining: Hematoxylin and Eosin (HE). Objective with 40× magnification. (**I**) Represents area of hepatic necrosis. (**J**) Represents the hepatocyte binucleation. (**K**) Represents the hyperemia index. Control group: no treatment; APAP group (500 mg/kg); Piperine-treated group (20 mg/kg); Piperine-treated group (40 mg/kg), NAC-treated group (300 mg/kg); NAC+P20 group, and NAC+P40 group. Results are expressed as median and interquartile range (n = 7). In graphs (**J**,**K**), contingency statistical analysis was evaluated by the chi-square test and Fisher’s exact test, with different letters indicating statistical differences between the groups. Differences were considered significant at *p* < 0.05. (+) Presence and (−) absence of paracetamol, piperine 20 mg/Kg, piperine 40 mg/Kg, N-acetylcysteine.

After observing an improvement in the liver function of the mice treated with the combination of piperine and NAC, we performed histological analyses to consolidate the hepatoprotective effects of the treatment with the combination of NAC and piperine. We evaluated the necrosis index (Figure 2I), binucleated hepatocytes (Figure 2J), and hyperemia index (Figure 2K).

Regarding necrosis in the centrilobular region of the liver (Figure 2, Figure 2I), the P40 (Figure 2D) and NAC (Figure 2E) groups showed a decreased area of necrosis. The NAC+P20 (Figure 2F) and NAC+P40 (Figure 2G) groups showed a marked reduction in the levels of necrosis when compared to the APAP group, and intact and well-defined hepatocytes were observed.

We assessed liver regeneration at the cellular level using semiquantitative binucleation analysis of hepatocytes (Figure 2J). We observed a significant increase in binucleated cells in the P40, NAC, NAC+P20, and NAC+P40 groups, suggesting a higher rate of regeneration in these groups than in the APAP group. In the APAP and P20 groups, there was a significant decrease in binucleated cells compared to the other groups. Analysis of hyperemia revealed that, compared to the control, P40, and NAC groups, the APAP, P20, NAC+P20, and NAC+P40 groups had an increased hyperemia index (Figure 2K), suggesting increased blood flow.

**Figure 3:**
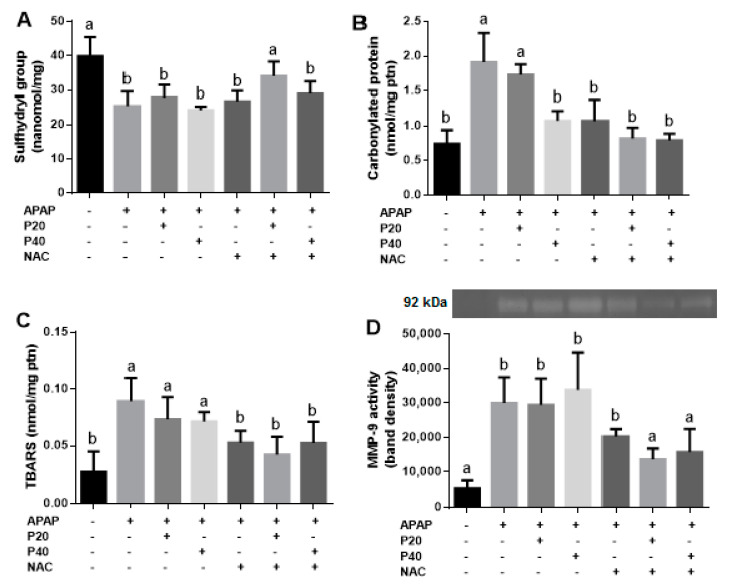
Evaluation of sulfhydryl group concentration (**A**). The levels of carbonylated protein (**B**). The levels of thiobarbituric acid-reactive substances (TBARS) (**C**). The enzymatic activity of matrix metalloproteinase (MMP-9) (**D**) in paracetamol intoxicated C57BL/6 mice. Statistical analysis was performed using one-way analysis of variance and Tukey’s post-test. Different letters (a, b) indicate significant difference between groups (*p* < 0.05), whereas the same letters indicate no significant difference between groups (*p* > 0.05). (+) Presence or (−) absence of paracetamol, piperine 20 mg/kg, piperine 40 mg/kg, N-acetylcysteine.

Knowing that piperine has antioxidant potential, we evaluated the cellular redox status, represented by the levels of sulfhydryl groups (Figure 3A), and the possible damage caused by protein and lipid oxidation, represented by the levels of carbonylated proteins (Figure 3B), TBARS (Figure 3C), and metalloproteinase 9 (MMP-9) activity (Figure 3D).

We determined the levels of sulfhydryl groups to evaluate the antioxidant profile of the experimental groups. We observed a reduction in the levels of the sulfhydryl groups in the APAP, P20, P40, NAC, and NAC+P40 groups compared to the control group. In the NAC+P20 group, no statistically significant difference was observed when compared to the control group, suggesting that this was the only group that maintained the antioxidant levels.

As shown in Figure 3B, we observed an increase in carbonylated proteins in the APAP, P20, and P40 groups compared to the control group. The groups treated with NAC, NAC+P20, and NAC+P40 showed a significant reduction when compared to the APAP group, which did not differ significantly from the control group. Regarding the lipid oxidation profile (Figure 3C), we observed an increase in TBARS in the APAP, P20, and P40 groups compared to the control group, which was similar to that observed in the oxidation of proteins, and the groups treated with NAC, NAC+P20, and NAC+P40 showed a reduction in TBARS compared to the APAP group, which did not differ significantly from the control group. In Figure 3D, we evaluated the activity of metalloproteinase-9 (MMP-9), which is an enzyme activated during hepatocyte damage. We observed that only the NAC+P20 and NAC+P40 groups had a significant reduction in MMP-9 activity compared to the APAP group.

**Figure 4:**
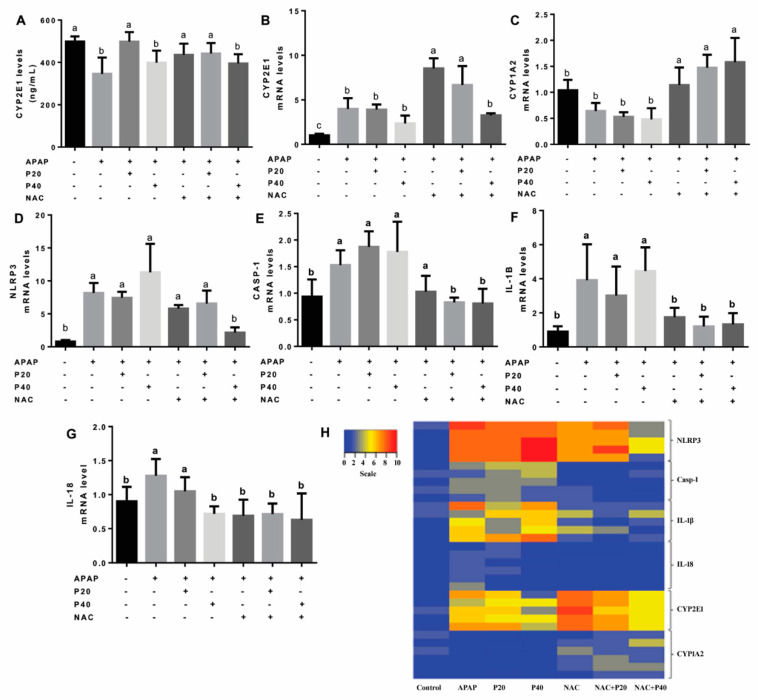
Levels of the CYP2E1 isoenzyme (**A**). Evaluation of the expression of CYP2E1 (**B**) and CYP1A2 (**C**) isoenzyme genes of cytochrome P450 and genes of the inflammasome pathway (**D**–**G**). Statistical analysis was performed using one-way analysis of variance and Tukey’s post-test. Different letters (a, b, c) indicate significant difference between groups (*p* < 0.05), whereas the same letters indicate no significant difference between groups (*p* > 0.05). (+) Presence or (−) absence of paracetamol, piperine 20 mg/kg, piperine 40 mg/kg, N-acetylcysteine. The mRNA heat map shows upregulated and downregulated genes with red and blue coloration, respectively (**H**). The genes evaluated were from the inflammasome pathway (*NLRP-3*, *Casp-1*, *IL-1β* and *IL-18*) and cytochrome P450 (*CYP2E1* and *CYP1A2*) (**B**). Abbreviation: CYP1A2: cytochrome P450 1A2; CYP2E1: cytochrome P450 2E1; Casp-1: caspase 1; IL-1β—interleukin 1β; IL-18: interleukin 18; and NLRP3: pyrin domain of the NLR family containing 3 proteins.

One of the main features of the mechanism of paracetamol-induced hepatotoxicity is the formation of a toxic metabolite produced by the cytochrome P450 system. Therefore, we evaluated the levels of cytochrome P450 through the CYP2E1 isoenzyme. After 12 h of APAP intoxication, we observed a reduction in the CYP2E1 level (Figure 4A), the main isoenzyme in the metabolism of paracetamol, in the APAP, P40, and NAC+P40 groups. In the groups treated with P20, NAC, and NAC+P20, the levels of CYP2E1 did not differ from that of the control group.

Figure 4 shows the expression of the *CYP2E1* (Figure 4G) and *CYP1A2* (Figure 4H) genes. We observed that the expression of the *CYP1A2* gene increased in the NAC, NAC+P20, and NAC+P40 treatments. Furthermore, the APAP, P20, P40, and NAC+P40 groups showed increased *CYP2E1* gene expression compared to the control group and decreased expression compared to the NAC and NAC+P20 groups.

Furthermore, we evaluated the expression of genes related to the inflammasome pathway (*NLRP3*, *CASP-1*, *IL-1β*, and *IL-18*) (Figure 4C–F). As shown in Figure 4C, there was a significant reduction in *NLRP3* gene mRNA levels only in the NAC+P40 group when compared to the other groups, and its expression did not differ from that of the control group. Regarding the expression of the *CASP-1* gene (Figure 4D), there was a significant reduction in the groups treated with NAC+P20 and NAC+P40 when compared to the APAP, P20, P40, and NAC groups. *IL-1β* gene expression (Figure 4E) was higher in the APAP, P20, and P40 groups than in the control group. In contrast, the NAC groups with or without P20 or P40 showed a reduction in *IL-1β* gene expression when compared to the APAP, P20, and P40 groups. In addition, we observed a reduction in the *IL-18* mRNA levels P40 and NAC groups with or without P20 and P40 when compared to the APAP and P20 groups (Figure 4F). The genes were compared using a heat map to evaluate the upregulated and downregulated genes (Figure 4B). We can qualitatively observe that in the control group, both the genes related to the inflammasome pathway and the genes related to cytochrome P450 were downregulated. In the APAP group, the inflammasome pathway and CYP2E1 genes were upregulated. In the NAC+P40 group, the genes related to the inflammasome and cytochrome P450 pathway were downregulated.

**Figure 5:**
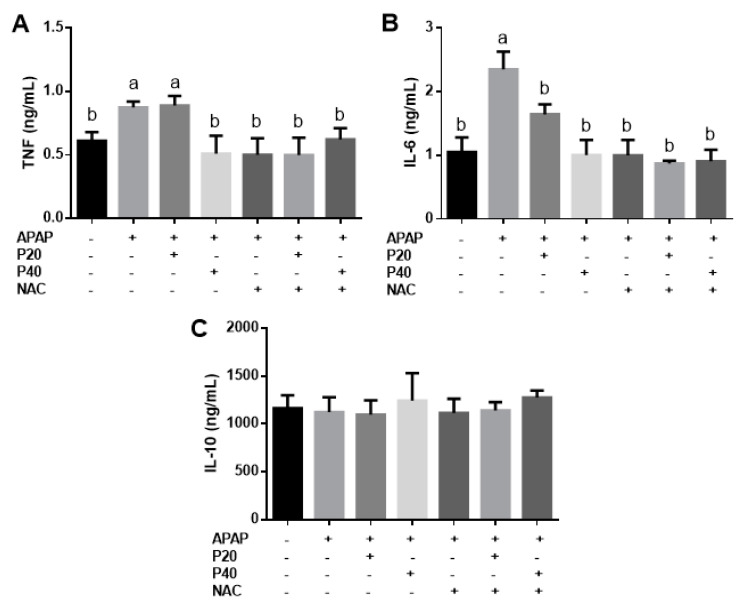
Evaluation of cytokines concentration in the serum: TNF (**A**); IL-6 (**B**); and IL-10 (**C**). Statistical analysis was performed using one-way analysis of variance and Tukey’s post-test. Different letters (a, b) indicate significant difference between groups (*p* < 0.05), whereas the same letters indicate no significant difference between groups (*p* > 0.05). (+) Presence or (−) absence of paracetamol, piperine 20 mg/kg, piperine 40 mg/kg, N-acetylcysteine. Abbreviation: TNF: tumor necrosis factor; IL-6: interleukin 6; IL-10: interleukin 10.

Regarding the serum inflammatory profile, we only observed an increase in TNF (Figure 5A) in the APAP and P20 groups. In the groups treated with P40, NAC, NAC+P20, and NAC+P40, the TNF levels were reduced compared to those in the APAP and P20 groups. There was a significant reduction in IL-6 (Figure 5B) in all the treatment groups compared to the APAP group. In addition, there was no statistical difference in the IL-10 levels (Figure 5C) between groups.

**Figure 6:**
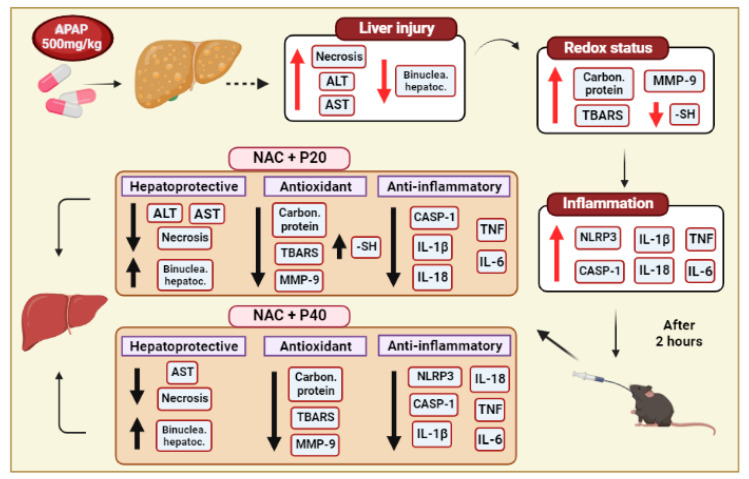
Representative drawing of the APAP-induced liver intoxication after 12 h and the treatments (NAC+P20 and NAC+P40). Paracetamol was administered at a dose of 500 mg/kg and the hepatic, histological, oxidative, and inflammatory changes are represented in the boxes and indicated with the red arrow. As treatment, we used piperine (P) associated or not associated with n-acetylcysteine (NAC) and the results are represented in the boxes and indicated with black arrow. Upward arrows indicate an increase and downward arrows indicate a decrease in the evaluated indexes. Abbreviations: paracetamol (APAP), N-acetylcysteine (NAC), piperine 20 mg/kg (P20), piperine 40 mg/kg (P40), alanine aminotransferase (ALT), aspartate aminotransferase (AST), carbonylated protein, thiobarbituric acid-reactive substances (TBARS), metalloproteinase-9 (MMP-9), sulfhydryl (-SH) group, caspase-1(casp-1), interleucina 1 beta (IL-1β), interleucina 18 (IL-18), interleucina 6 (IL-6), and tumor necrosis factor (TNF).

The treatments performed after 12 h of paracetamol intoxication are summarized in the representative design in Figure 6.

## 4. Discussion

APAP overdose can cause liver necrosis, redox imbalance, and increased inflammatory cytokines [33]. Our data corroborate with those of others, demonstrating that APAP overdose leads to liver damage [25,34]. NAC is known to be the only therapeutic option for APAP overdose; however, this drug has its limitations, including adverse effects and a narrow therapeutic window [35]. Therefore, studies aimed at evaluating drug repositioning to improve the efficacy and therapeutic time of NAC are important. In recent years, the protective effects of natural products against APAP-induced hepatotoxicity have been studied [36]. In this direction, piperine has been considered for the treatment of APAP overdose, since it has antioxidant [15] and anti-inflammatory properties [37]. One of the most critical parameters of APAP intoxication is the death of hepatocytes as a result of necrosis, and our data showed that the area of liver necrosis was reduced following treatment with two different doses of piperine (20 or 40 mg/kg) in combination with NAC, increasing the efficacy of this drug. The treatments with NAC with or without 20 or 40 mg/kg piperine was also effective in reducing AST, whereas the treatment with NAC in combination with 20 mg/kg piperine was effective in reducing ALT. Thus, it is possible that the hepatoprotective capacity of piperine already described in the literature [15,38] may potentiate the beneficial effects of NAC in the treatment of paracetamol intoxication.

The proliferation of liver cells from binucleated hepatocytes indicates cell turnover; thus, our results demonstrate that treatment with 40 mg/kg piperine or NAC with or without piperine (20 or 40 mg/kg) increased the binucleation index, indicating the regenerative capacity of hepatocytes. The increase in hyperemia may be related to both vasodilation and accumulation of red blood cells induced by the inflammatory process [39]. Thus, the cause of increase in hyperemia in the APAP group is different from the P20, NAC+P20, and NAC+P40 groups. The first case was attributed to the inflammatory process characteristic of intoxication with APAP, and the second case demonstrated the ability of piperine to act as a physiological vasodilator [40]. Results demonstrate that piperine induces vasodilatory effects in the aorta of rats [41], which may be one of the factors responsible for favoring synergy with the increased bioavailability of NAC in the piperine-associated groups.

It has been shown that APAP intoxication induces redox imbalance and oxidative damage [35]. In our study, we observed that TBARS and carbonylated protein levels were reduced in the groups treated with NAC with or without piperine. In addition, the 40 mg/kg piperine group showed also a reduction in carbonylated protein levels compared to the APAP group. These results are in agreement with those of other studies suggesting that piperine protects against oxidative damage to proteins and lipids [42,43].

The antioxidant defense levels provided by the availability of glutathione to eliminate NAPQI upon APAP overdose were evaluated based on the concentration of the sulfhydryl groups. Only the treatment with NAC plus 20 mg/kg piperine was able to increase the levels of -SH. It has been suggested that the hepatoprotective effect of piperine may be mediated through its antioxidant potential, the reduction in lipid peroxidation, and the intensification of the antioxidant defense system [13,44]. The antioxidant potential of NAC has been confirmed in various studies; however, the ability of piperine to potentiate the effects of controlling changes in redox state demonstrates the improved efficacy of the drug. Notably, a 20 mg/kg dose of piperine showed a greater and more beneficial synergistic effect on most parameters.

APAP intoxication induces activation of matrix metalloproteinase 9 (MMP-9), a gelatinase involved in extracellular matrix (ECM) degradation, increasing leukocyte infiltration and inflammatory status, and is detrimental to liver function [45]. Previous studies reported the role of MMP-9 in the induction of liver damage and microcirculation dysfunction in animals intoxicated with APAP [34]. It has been shown that MMP-9 activity modifies vascular integrity and liver regeneration, suggesting that MMP-9 inhibition may be considered an important target for regulating liver inflammation and damage [46,47]. We confirmed that only the piperine group (20 and 40 mg/kg) associated with NAC significantly reduced gelatinase levels. This suggests that the combination of NAC and piperine exerts a hepatoprotective mechanism.

The metabolism of high-dose APAP in the liver depends on CYP450 isoenzymes, which are responsible for the oxidation of the drug and the formation of the reactive metabolite NAPQI [48,49]. Increased *CYP2E1* mRNA expression may contribute to APAP-induced hepatotoxicity [3]. However, inhibition of CYP2E1 by APAP, as demonstrated in our APAP group, has been reported in previous studies, even at lower doses than those used in our study (300 mg/kg APAP) after 12 h of intoxication [50]. Thus, APAP may decrease CYP2E1 activity in vivo to counter-regulate the formation of toxic NAPQI [50,51]. The increase in *CYP2E1* mRNA expression in these groups may be related to piperine metabolism via the same pathway as APAP [19]. Similar to other natural compounds containing methylenedioxyphenyl substituents, piperine affects cytochrome P450 isoforms and may inhibit or activate cytochrome, depending on the CYP isoform and the dose of piperine used [52]. A lower dose of piperine may be metabolized by CYP2E1, thus acting as a competitor for APAP metabolism.

Studies have demonstrated the immunomodulatory potential of piperine [37]; therefore, our next step was to evaluate the expression of inflammasome pathway genes and serum cytokines. It is known that the inflammasome complex contributes to the aggravation of APAP-induced liver injury [53]. NLRP-3 activation is associated with advanced stages of APAP intoxication [54]. We observed a significant increase in NLRP-3 gene expression in the APAP group and a reduction in its expression only in the group treated with NAC+P40. A previous study with piperine demonstrated its ability to reduce NLRP-3 gene expression in the kidneys of diabetic rats at a dose of 30 mg/kg [55]. The expression of *CASP-1*, which is activated by NLRP-3, was reduced in the NAC+P20 and NAC+P40 groups. We also observed that expression *IL-1β* was reduced in NAC groups with or without piperine and expression of *IL-18* was reduced in the P40, NAC, NAC+P20, and NAC+P40 groups compared to the APAP group. Thus, piperine in combination with NAC may have contributed to the decreased expression of components of the inflammasome pathway and was shown to enhance the immunoregulatory role by reducing the activation of this pathway, which may be an important factor in reducing liver injury in APAP intoxication.

The pro-inflammatory cytokines IL-6 and TNF are important mediators of inflammation activation and pathological progression in APAP-induced hepatotoxicity [50,51]. We observed that TNF was produced upon APAP intoxication and was able to stimulate the secretion of IL-6 [56]. Our results showed that IL-6 was reduced in all treated groups and TNF was reduced in the P40, NAC, NAC+P20, and NAC+P40 groups. Correspondingly, others have also demonstrated that piperine reduces pro-inflammatory factors such as TNF and IL-6 [37]. The reduction in TNF following piperine treatment indicates that it can reduce inflammation [21]. IL-6 plays an important role in neutrophil-mediated inflammation and is activated in response to tissue injury and infection [57,58]. The reduction of TNF and IL-6 suggests the anti-inflammatory efficacy of piperine and may be an indication of reduced cell death in the liver.

## 5. Conclusions

The association of piperine and NAC effectively reduced liver injury, as observed by the reduction in necrosis and oxidative damage biomarkers, the downregulation of the inflammasome pathway, and the reduction in serum TNF and IL-6 levels. In addition, the association of NAC with the lowest dose of piperine reduced serum ALT levels and increased hepatic thiol levels. Therefore, the therapeutic effects of NAC can be potentiated by piperine to treat paracetamol-induced hepatotoxicity.

## Data Availability

The data presented in this study are available in the article text.
